# Supplementary material for: The Influence of Food Waste Rearing Substrates on Black Soldier Fly Larvae Protein Composition: A Systematic Review
Source: Insects. 2021 Jul 4;12(7):608. doi: 10.3390/insects12070608 (PMC8303580; doi:10.3390/insects12070608)
Supplement: Supplementary file 1 [file insects-12-00608-s001.zip › insects-1217248-supplementary.pdf]

insects MDPI

		Essential Amino Acids									Non-essential Amino Acids								
Author	Rearing Substrate (RS) *(mixture ratio)*	Histidine (%)	Isoleucine (%)	Leucine (%)	Lysine (%)	Methionine (%)	Phenylalanine (%)	Threonine (%)	Tryptophan (%)	Valine (%)	Alanine (%)	Arginine (%)	Aspartate (%)	Cysteine (%)	Glutamate (%)	Glycine (%)	Proline (%)	Serine (%)	Tyrosine (%)
Liland [26]	RS 1: Wheat	2.7	4.0	7.7	5.6	1.7	4.6	4.3	-	5.9	6.3	6.0	8.7	-	19.1	6.4	7.6	5.5	3.9
	RS 2: Wheat, brown algae *A. nodosum* (9:1)	2.6	4.0	7.6	5.8	1.6	4.4	4.4	-	5.9	6.5	5.7	9.2	-	20.2	6.1	7.2	5.4	3.4
	RS 3: Wheat, brown algae *A. nodosum* (8:2)	2.6	4.0	7.5	5.5	1.7	4.5	4.4	-	5.8	6.3	5.8	9.1	-	20.3	6.3	7.0	5.5	3.7
	RS 4: Wheat, brown algae *A. nodosum* (7:3)	2.5	3.9	7.5	5.6	1.6	4.3	4.5	-	5.7	6.5	5.5	9.7	-	20.8	6.1	6.8	5.5	3.4
	RS 5: Wheat, brown algae *A. nodosum* (6:4)	2.5	4.0	7.4	5.3	1.7	4.7	4.6	-	5.7	6.4	5.5	9.7	-	20.6	6.3	6.6	5.5	3.6
	RS 6: Wheat, brown algae *A. nodosum* (5:5)	2.3	3.9	7.3	5.4	1.7	4.4	4.6	-	5.6	6.6	5.2	10.3	-	21.5	6.1	6.3	5.5	3.2
	RS 7: Wheat, brown algae *A. nodosum* (4:6)	2.2	4.0	7.2	5.5	1.7	4.4	4.7	-	5.7	6.6	5.4	11.0	-	20.9	6.1	5.9	5.2	3.6
	RS 8: Wheat, brown algae *A. nodosum* (3:7)	2.2	4.0	7.2	5.4	1.7	4.6	4.7	-	5.7	6.7	5.4	11.1	-	20.9	6.0	5.8	5.0	3.5
	RS 9: Wheat, brown algae *A. nodosum* (2:8)	1.8	3.8	6.9	5.4	1.7	4.0	4.8	-	5.3	7.0	4.8	11.9	-	23.6	5.7	5.1	5.3	2.9
	RS 10: Wheat, brown algae *A. nodosum* (1:9)	1.5	3.8	6.8	5.2	1.8	4.2	4.8	-	5.2	7.2	4.5	12.8	-	24.9	5.5	4.4	4.8	2.7
	RS 11: Brown algae *A. nodosum*	1.1	3.8	6.7	5.1	1.9	4.2	4.8	-	5.1	7.3	4.4	13.6	-	25.9	5.3	3.7	4.6	2.5
Spranghers^ [33]	RS 1: Restaurant waste—potato, rice, pasta, vegetable *(ratio unspecified)*	0.4	0.6	1.1	0.7	0.3	0.7	0.6	0.2	0.7	0.7	0.7	1.5	0.2	3.3	0.6	1.1	0.7	-

Supplementary Table 1. Amino Acid Profile of Rearing Substrates.
Spranghers et al. [33] presented as a percentage of crude protein rearing substrate content. Liland et al. [26] presented as percentage of total sum of amino acids. ^ indicative of original article results presenting data as g/kg of crude protein of rearing substrate content. Dashes used to indicate where data is unreported in the original article.

	Sequence generation	Baseline characteristics	Allocation concealment	Random housing	Random outcome assessment	Incomplete outcome data	Selective outcome reporting	Other sources of bias
Barbi [19]	?	?	?	?	?	+	+	?
Barragán-Fonseca [20]	?	+	?	+	?	+	-	?
Bava [21]	?	?	?	?	?	+	-	?
Chia [22]	?	?	?	?	?	+	+	?
Danieli [23]	?	+	?	?	?	+	+	-
Ewald [24]	?	+	?	-	?	+	+	-
Gold [25]	?	+	?	?	?	+	+	?
Liland [26]	?	?	?	?	?	+	-	?
Liu [27]	?	+	?	?	?	-	+	?
Lopes [28]	?	?	?	?	?	+	+	?
Meneguz [29]	?	?	?	?	+	+	+	?
Oonincx [30]	?	+	?	?	?	+	+	-
Salomone [31]	?	?	?	?	?	+	+	-
Shumo [32]	?	+	?	?	?	+	+	?
Spranghers [33]	?	+	?	?	?	+	+	?
Tinder [34]	?	+	?	?	?	+	+	?
Tschirner [35]	?	+	?	+	?	+	-	?
Cappellozza [36]	?	+	?	?	?	+	+	-
Jucker [37]	?	+	?	?	?	+	+	-
Lalander [38]	?	+	?	?	?	-	-	?
Nguyen [39]	?	+	?	?	?	-	-	?
Barroso [40]	?	+	?	?	?	-	+	-
Surendra [41]	?	?	?	?	?	+	+	-

Author	Rearing Substrate (RS) (mixture ratio)	Development time (days)	Harvest stage	Survival rate (%)	Individual larval weight (mg)
Barbi ^ [19]	RS 1: Exotic fruit, melon (5:5)	29.0	100% prepupae	95.0	89.5
	RS 2: Exotic fruit, pineapple, kiwi, apple, melon (1:1:6:1:1)	29.0		83.3	66.5
	RS 3: Pineapple	33.7		60.3	78.3
	RS 4: Melon	24.0		99.3	74.1
	RS 5: Apple	25.3		89.3	66.5
	RS 6: Exotic fruit	31.3		59.0	60.1
	RS 7: Exotic fruit, pineapple, kiwi, apple, melon (2:2:2:2:2)	26.7		93.7	76.6
	RS 8: Exotic fruit, kiwi (5:5)	25.3		64.7	64.3
	RS 9: Pineapple, melon (5:5)	29.0		89.7	83.9
	RS 10: Kiwi, melon (5:5)	26.7		81.3	70.5
	RS 11: Pineapple, apple (5:5)	28.0		91.3	75.0
	RS 12: Exotic fruit, pineapple, kiwi, apple, melon (1:1:1:6:1)	28.7		87.7	74.5
	RS 13: Apple, melon (5:5)	26.3		94.3	85.7
	RS 14: Exotic fruit, pineapple, kiwi, apple, melon (1:1:1:1:6)	25.3		94.0	88.2
	RS 15: Pineapple, kiwi (5:5)	27.7		67.0	75.3
	RS 16: Exotic fruit, pineapple (5:5)	25.3		83.7	66.8
	RS 17: Kiwi	25.3		56.7	51.8
	RS 18: Exotic fruit, apple (5:5)	29.0		79.7	68.9
	RS 19: Exotic fruit, pineapple, kiwi, apple, melon (6:1:1:1:1)	32.3		87.0	65.1
	RS 20: Exotic fruit, pineapple, kiwi, apple, melon (1:1:1:1:6)	26.7		80.3	78.7
	RS 21: Kiwi, apple (5:5)	28.0		80.0	69.1
	RS 22: Peach, tomato (6.7:3.3)	26.0		93.0	141.6
	RS 23: Peach	33.0		87.3	139.9
	RS 24: All-year mix, peach, tomato (6.7:1.6:1.7)	20.3		93.0	132.4
	RS 25: All-year mix, peach, tomato (3.4:3.3:3.3)	21.3		99.3	132.4
	RS 26: All-year mix, tomato (3.3:6.7)	18.0		97.0	146.0
	RS 27: All-year mix, peach (6.7:3.3)	23.7		93.7	129.3
	RS 28: All-year mix, peach, tomato (1.7:6.7:1.6)	25.0		89.0	139.6
	RS 29: All-year mix, tomato (6.7:3.3)	18.0		94.7	128.4
	RS 30: All-year mix	18.0		91.7	127.5
	RS 31: Peach, tomato (3.3:6.7)	18.0		96.0	145.5
	RS 32: All-year mix, peach, tomato (1.6:1.7:6.7)	18.0		95.0	143.8
	RS 33: Tomato	18.0		99.0	169.9
	RS 34: All-year mix, peach (3.3:6.7)	33.0		94.3	139.7
	RS 35: Legume, corn, pomace, all-year mix (1.25:6.25:1.25:1.25)	14.0		98.7	212.3
	RS 36: Corn, all-year mix (5:5)	13.0		99.0	226.8
	RS 37: Corn	13.0		93.7	183.1
	RS 38: Legume, corn, pomace, all-year mix (1.25:1.25:6.25:12.5)	19.0		94.7	186.1
	RS 39: Legume, corn, pomace, all-year mix (6.25:1.25:1.25:1.25)	13.0		98.3	206.5
	RS 40: Legume	14.0		90.0	177.5
	RS 41: Legume, pomace (5:5)	15.0		98.3	188.9
	RS 42: All-year mix	19.0		99.0	136.8
	RS 43: Legume, corn (5:5)	12.0		93.7	192.9

	RS 44: Pomace, all-year mix (5:5)	26.0		93.3	144.8
	RS 45: Legume, corn. pomace, all-year mix (1.25:1.25:1.25:6.25)	14.0		99.3	186.5
	RS 46: Pomace	47.0		62.3	111.1
	RS 47: Corn, pomace (5:5)	15.0		96.0	203.6
	RS 48: Legume, all-year mix (5:5)	13.0		99.0	191.5
	RS 49: Legume, corn, pomace, all-year mix (2.5:2.5:2.5:2.5)	12.0		100.0	218.8
Barragán-Fonseca^ [20]	RS 1: High protein—dried distillers' grains with soluble, cabbage leaves, old bread, cellulose, sunflower oil (unspecified ratio)	22.0	50% prepupae	93.7	39.0
	RS 2: Low protein—dried distillers' grains with soluble, cabbage leaves, old bread, cellulose, sunflower oil (unspecified ratio)	21.6		75.7	42.0
Bava^ [21]	RS 1: Okara	-	40% prepupae	98.5	138.0
	RS 2: Maize distillers	16.0		73.0	197.0
	RS 3: Brewers' grains	22.0		95.9	98.0
Chia [22]	RS 1: Spent barley	-	5th instar	-	-
	RS 2: Spent barley, brewer's yeast	-		-	-
	RS 3: Spent barley, brewer's yeast, molasses	-		-	-
	RS 4: Spent malted barley	-		-	-
	RS 5: Spent malted barley, brewer's yeast	-		-	-
	RS 6: Spent malted barley, brewer's yeast, molasses	-		-	-
	RS 7: Spent malted corn	-		-	-
	RS 8: Spent malted corn, brewer's yeast	-		-	-
	RS 9: Spent malted corn, brewer's yeast, molasses	-		-	-
	RS 10: Spent sorghum, barley	-		-	-
	RS 11: Spent sorghum, barley, brewer's yeast	-		-	-
	RS 12: Spent sorghum, barley, brewer's yeast, molasses	-		-	-
Danieli ^ WW [23]	RS 1: Control—corn, wheat bran, dehydrated alfalfa (5:2:3)	21.0	70% prepupae	-	-
	RS 2: High non-fibre carbohydrate—ground barley, wheat bran, dehydrated alfalfa (6.8:2:1.2)	21.0		-	-
	RS 3: High fibre carbohydrate—ground barley, wheat middlings, dehydrated alfalfa, wheat straw (1.6:5:1:2.4)	21.0		-	-
	RS 4: High protein—ground barley, wheat middlings, dehydrated alfalfa (1.5:5.5:3)	21.0		-	-
Ewald WW [24]	RS 1: Bread	14.0	-	69.8	137.0
	RS 2: Fish *O. mykiss*, wheat (5:1)	14.0		18.4	89.0
	RS 3: Food waste (uncharacterised)	14.0		89.1	191.0
	RS 4: Fresh mussels *M. edulis*	14.0		89.3	235.0
	RS 5: Bread, fresh mussels *M. edulis* (9:1)	14.0		86.2	133.0
	RS 6: Bread, fresh mussels *M. edulis* (8:2)	14.0		82.1	181.0
	RS 7: Bread, fresh mussels *M. edulis* (7:3)	14.0		68.0	168.0
	RS: 8 Bread, fresh mussels *M. edulis* (6:4)	14.0		77.1	138.0
	RS: 9 Bread, fresh mussels *M. edulis* (5:5)	14.0		54.3	131.0
Gold [25]	RS 1: Mill by-products	9.0	-	96.2	41.7

	RS 2: Canteen waste– mix of vegetables with/without dressing, sausage, offal (ratio unspecified)	9.0		92.3	44.2
	RS 3: Poultry waste	9.0		90.7	39.4
	RS 4: Vegetable canteen waste—mix of vegetables with/without dressing (ratio unspecified)	9.0		97.5	59.1
	RS 5: Mixed food waste—(1:1:1 of RS1:RS2:RS3)	9.0		97.0	62.8
Liland [WW] [26]	RS 1: Wheat	8.0	-	>95.0	135.0
	RS 2: Wheat, brown algae *A. nodosum* (9:1)	8.0		>95.0	-
	RS 3: Wheat, brown algae *A. nodosum* (8:2)	8.0		>95.0	-
	RS 4: Wheat, brown algae *A. nodosum* (7:3)	8.0		>95.0	-
	RS 5: Wheat, brown algae *A. nodosum* (6:4)	8.0		>95.0	-
	RS 6: Wheat, brown algae *A. nodosum* (5:5)	8.0		>95.0	-
	RS 7: Wheat, brown algae *A. nodosum* (4:6)	8.0		>95.0	-
	RS 8: Wheat, brown algae *A. nodosum* (3:7)	8.0		>95.0	-
	RS 9: Wheat, brown algae *A. nodosum* (2:8)	8.0		82.0	-
	RS 10: Wheat, brown algae *A. nodosum* (1:9)	8.0		76.0	-
	RS 11: Brown algae *A. nodosum*	8.0		51.0	28.0
Liu [27]	RS 1: Brewery by-product (uncharacterised)	15.0	100% prepupae	98.0	45.5
Lopes [28]	RS 1: Bread	12.0	1% prepupae	88.5	116.5
	RS 2: Bread, fish *O. mykiss*, (9.5:0.5)	11.0		81.6	149.9
	RS 3: Bread, fish *O. mykiss*, (9:1)	11.0		76.4	146.7
	RS 4: Bread, fish *O. mykiss*, (8.5:1.5)	12.0		65.4	157.5
Meneguz^ [29]	RS 1: Fruit and vegetable mix—celery, oranges, peppers (4.3:2.9:2.8)	20.2	30% prepupae	88.8	120.0
	RS 2: Fruit—apples, oranges, apple leftovers, strawberries, mandarins, pears, kiwis, bananas, lemons (4.8:1.5:1.4:0.7:0.5:0.4:0.3:0.2:0.2)	22.0		80.7	180.0
	RS 3: Winery by-products—grape seeds, pulp, skins, stems, leaves (ratio unspecified)	22.2		75.2	150.0
	RS 4: Brewery by-products—barley brewers' grains	8.0		90.5	120.0
Oonincx [30]	RS 1: High protein high fat—spent grains, beer yeast, cookie remain (6:2:2)	21.0	First prepupae	86.0	-
	RS 2: High protein low fat—beer yeast, potato steam peelings, beet molasses (5:3:2)	33.0		77.0	-
	RS 3: Low protein high fat—cookie remains, bread (5:5)	37.0		72.0	-
	RS 4: Low protein low fat—potato steam peelings, beet molasses, bread (3:2:5)	37.0		74.0	-
Salomone [31]	RS 1: Food waste—vegetable, meat/fish, bread/pasta/rice, other (6.5:0.5:2.5:0.5)	12.0	-	-	-
Shumo [32]	RS 1: Kitchen waste—potato peelings, carrot, rice, bread debris (ratio unspecified)	-	-	-	-

	RS 2: Brewery by-product	-		-	-
Spranghers [33]	RS 1: Restaurant waste—potato, rice, pasta, vegetable (ratio unspecified)	19.0	6 days after first prepupae	-	-
Tinder [34]	RS 1: Sorghum	38.1	40% prepupae	50.8	145.0
	RS 2: Sorghum, cowpea (7.5:2.5)	32.2		41.5	151.8
	RS 3: Sorghum, cowpea (5:5)	28.2		43.1	156.7
	RS 4: Sorghum, cowpea (2.5:7.5)	28.4		40.3	254.3
	RS 5: Cowpea	29.7		44.3	137.9
	RS 6: Sorghum-B	38.1		33.9	95.3
	RS 7: Sorghum, cowpea (7.5:2.5)-B	35.3		32.0	91.6
	RS 8: Sorghum, cowpea (5:5)-B	35.1		23.1	101.0
	RS 9: Sorghum, cowpea (2.5:7.5)-B	15.8		25.0	92.1
	RS 10: Cowpea-B	34.8		32.7	101.0
Tschirner WW [35]	RS 1: Carbohydrate—wheat middlings	15.0	-	55.6	290.0
	RS 2: Protein—dried distillers' grains with soluble	15.0		21.7	271.0
	RS 3: Fibre—sugar beet	15.0		78.3	35.0
Cappellozza [36]	RS 1: Fruit and vegetable mix—zucchini, apple, potato, green beans, carrot, pepper, orange, celery, kiwi, plum, eggplant (unspecified ratio)	31.0	40% prepupae	-	231.0
Jucker ^ FW [37]	RS 1: Fruit—apple, pear, orange (3.3:3.3:3.3)	52.0	40% prepupae	96.4	174.0
	RS 2: Vegetable—lettuce, string green beans, cabbage (3.3:3.3:3.3)	48.3		88.7	184.0
	RS 3: Fruit and vegetable mix—(1:1 of RS1:RS2)	36.7		93.7	154.0
Lalander [38]	RS 1: Food waste (uncharacterised)	19.0	50% prepupae	87.2	212.0
	RS 2: Fruit and vegetable mix—lettuce, apple, potato (5:3:2)	42.0-47.0		90.7	218.0
Nguyen [39]	RS 1: Kitchen waste (animal and plant matter)	-	-	-	227.0
	RS 2: Fruit and vegetable (uncharacterised)	-		-	170.0
	RS 3: Fish (uncharacterised)	-		-	167.0
Barroso [40]	RS 1: Fish waste *S. aurita* - reared 1 day	1.0	-	-	-
	RS 2: Fish waste *S. aurita* - reared 2 day	2.0		-	-
	RS 3: Fish waste *S. aurita* - reared 4 day	4.0		-	-
	RS 4: Fish waste *S. aurita* - reared 6 day	6.0		-	-
	RS 5: Fish waste *S. aurita* - reared 8 day	8.0		-	-
	RS 6: Fish waste *S. aurita* - reared 10 day	10.0		-	-
	RS 7: Fish waste *S. aurita* - reared 12 day	12.0		-	-
Surendra [41]	RS 1: Food waste (uncharacterised)	-	-	-	-

Supplementary Table 3. Life history traits of Black Soldier Fly Larvae Reared on Food Waste.

Results presented as means. ^ indicative of original article presenting weight data as grams. mg, milligrams, DM dry matter, WW wet weight., FW fresh weight.
